# The role of transcription bodies in gene expression: what embryos teach us

**DOI:** 10.1042/BST20240599

**Published:** 2025-02-06

**Authors:** Martino Ugolini, Nadine L. Vastenhouw

**Affiliations:** Center for Integrative Genomics (CIG), University of Lausanne (UNIL), Lausanne, Switzerland

**Keywords:** Transcription bodies, Gene regulation, Developmental model systems, Nuclear organization, Sequestration

## Abstract

Transcription does not occur diffusely throughout the nucleus but is concentrated in specific areas. Areas of accumulated transcriptional machinery have been called clusters, hubs, or condensates, while transcriptionally active areas have been referred to as transcription factories or transcription bodies. Despite the widespread occurrence of transcription bodies, it has been difficult to study their assembly, function, and effect on gene expression. This review highlights the advantages of developmental model systems such as zebrafish and fruit fly embryos, in addressing these questions. We focus on three important discoveries that were made in embryos. (i) It had previously been suggested that, in transcription bodies, the different steps of the transcription process are organized in space. We explore how work in embryos has revealed that they can also be organized in time. In this case, transcription bodies mature from transcription factor clusters to elongating transcription bodies. This type of organization has important implications for transcription body function. (ii) The relevance of clustering for *in vivo* gene regulation has benefited greatly from studies in embryos. We discuss examples in which transcription bodies regulate developmental gene expression by compensating for low transcription factor concentrations and low-affinity enhancers. Finally, (iii) while accumulations of transcriptional machinery can facilitate transcription locally, work in embryos showed that transcription bodies can also sequester the transcriptional machinery, modulating the availability for activity at other sites. In brief, the reviewed literature highlights the properties of developmental model organisms that make them powerful systems for uncovering the form and function of transcription bodies.

## Introduction

An important question in biology is how cells control which genes are to be turned on and which genes are to be turned off. Deciphering the mechanisms that regulate gene expression could potentially help to treat diseases linked to the misregulation of genes and to harness the regenerative capabilities of cells. Despite considerable progress over the years, our knowledge of gene expression regulation remains incomplete.

**Figure 1:**
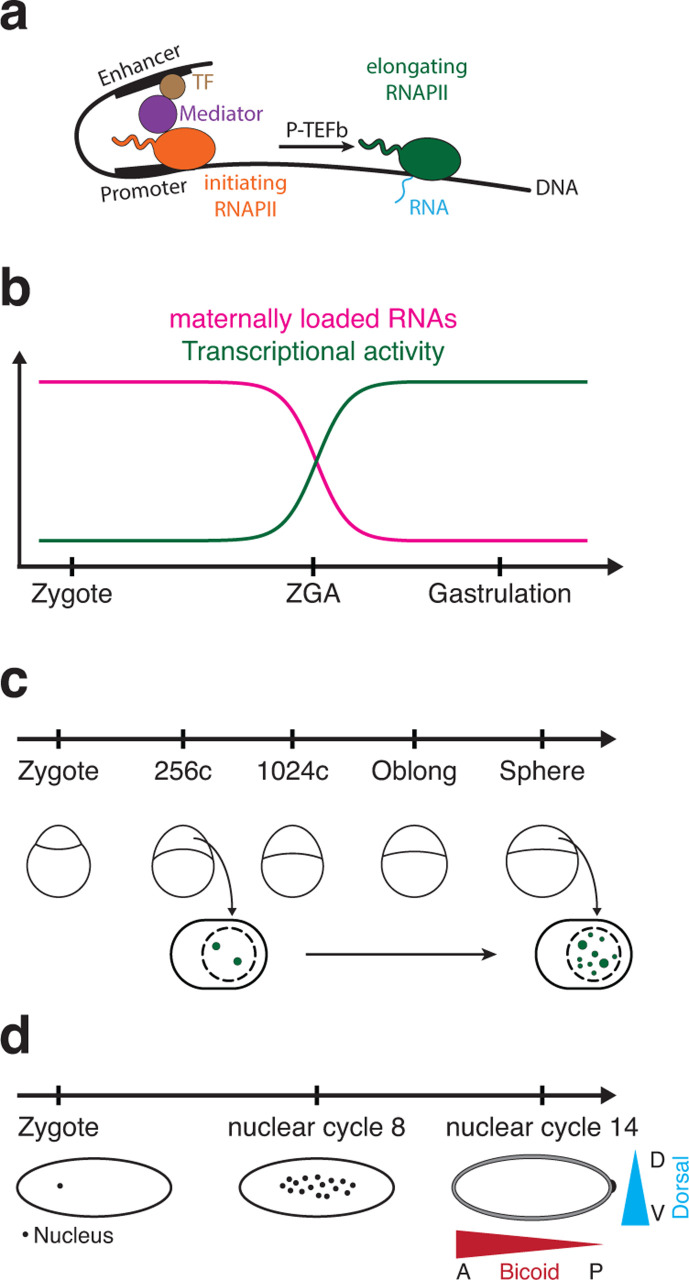
Zebrafish and fruit fly embryos provide powerful models to study the role of transcription bodies in gene expression regulation. **(a)** A simplified model of the transcription process. TF, transcription factor. **(b)** During zygotic genome activation (ZGA), embryos gradually transition from a state of transcriptional silence to full transcriptional activity in only a few cell cycles. In parallel, maternally provided factors that drive development in the early stages are degraded. **(c)** During zebrafish ZGA, transcriptional elongation (in green) is initially concentrated in two very large and long-lived transcription bodies. These have been used to determine the form and function of transcription bodies. **(d)** During early development in *Drosophila*, the body plan of the embryo is established through multiple transcription factor gradients, including the factors Bicoid (anterior/posterior (A/P) gradient) and Dorsal (dorsal/ventral (D/V) gradient). Bicoid and Dorsal form transcription factor clusters and have well-defined target enhancers and target genes. These properties have been important to establish the role of transcription factor clustering in gene expression regulation and development.

Transcription is a multistep process. In the simplest form, transcription of a gene is driven by just a promoter. When an enhancer is involved, a transcription factor binds to an enhancer, which – through the Mediator complex – contacts the promoter, resulting in the recruitment of RNA polymerase II (RNAPII) and the activation of transcription (Figure 1a). RNAPII is initially stalled at the promoter in the initiation state, only to be released to enter the elongation phase later. The transition from initiation to elongation is highly regulated and is ultimately mediated by the P-TEFb complex and its kinase CDK9 [1].

In the 1990s, cell biologists observed that transcription does not occur diffusely throughout the nucleus but is highly organized in nuclear space. With the advances in microscopy, the accumulation of transcriptional machinery such as transcription factors and RNA pol II has been widely observed [2–5] and has been referred to as clusters, hubs, or condensates. Areas of transcriptional activity have been referred to as transcription factories or transcription bodies. These – logically – often coincide with accumulations of transcriptional machinery. Because, especially, the terms condensate and transcription factory are associated with specific models regarding the formation and function of accumulation, we use the more descriptive term transcription body.

Electron microscopy studies have suggested that most transcriptional activity in the nucleus occurs in transcription bodies [6]. Often, transcription bodies are not simply the result of individual highly expressed genes but are rather hubs in which multiple genes are expressed [7–10]. Transcription bodies can be specialized, expressing, for example, only genes that require a specific polymerase [11], that share the same promoter [12], or that are regulated by shared enhancers [13]. Nonetheless, it has long remained unclear how transcription bodies assemble, what function they have in gene regulation, and how they interact with their target genes.

These questions are hard to address in cultured cells because transcription bodies are often highly abundant (100–2000 per cell [7]), small (40–80 nm in diameter [2,6,14–16]), and short-lived (less than a minute [3,17]). In addition, the biological relevance of transcription bodies is difficult to assess in systems in which clear phenotypic outputs are lacking. For these reasons, the field has benefited greatly from the use of classical models in developmental genetics such as *Danio rerio* (zebrafish) and *Drosophila melanogaster* (fruit fly) embryos. Early development of these organisms is characterized by the stereotyped and well-characterized onset of transcription during a process called zygotic genome activation (ZGA) [18–20] (Figure 1b). At the onset of transcription, the nucleus is devoid of heterochromatin, and over time, it transitions into a fully matured and transcriptionally active nucleus. In zebrafish embryos, transcription starts in two large transcription bodies that are isolated, relatively large (microns in diameter), and long-lived (minutes) [21–23] (Figure 1c). The simple nuclear organization that characterizes the onset of transcription in zebrafish embryos has proven very useful in addressing the assembly and function of transcription bodies. In addition, the gradual activation of transcription during ZGA is deeply linked with clearly observable phenotypes. Especially in the fruit fly embryo, which has a set of well-characterized transcription factor gradients (Figure 1d) [24], this has allowed researchers to investigate the importance of transcription bodies in developmental gene expression.

Here, we describe three important features of transcription bodies that have been studied in embryonic systems: (i) their dynamic formation, (ii) their effect on developmental gene expression, and (iii) their role in regulating the availability of transcriptional machinery.

### Transcription bodies assemble in a sequential manner

**Figure 2:**
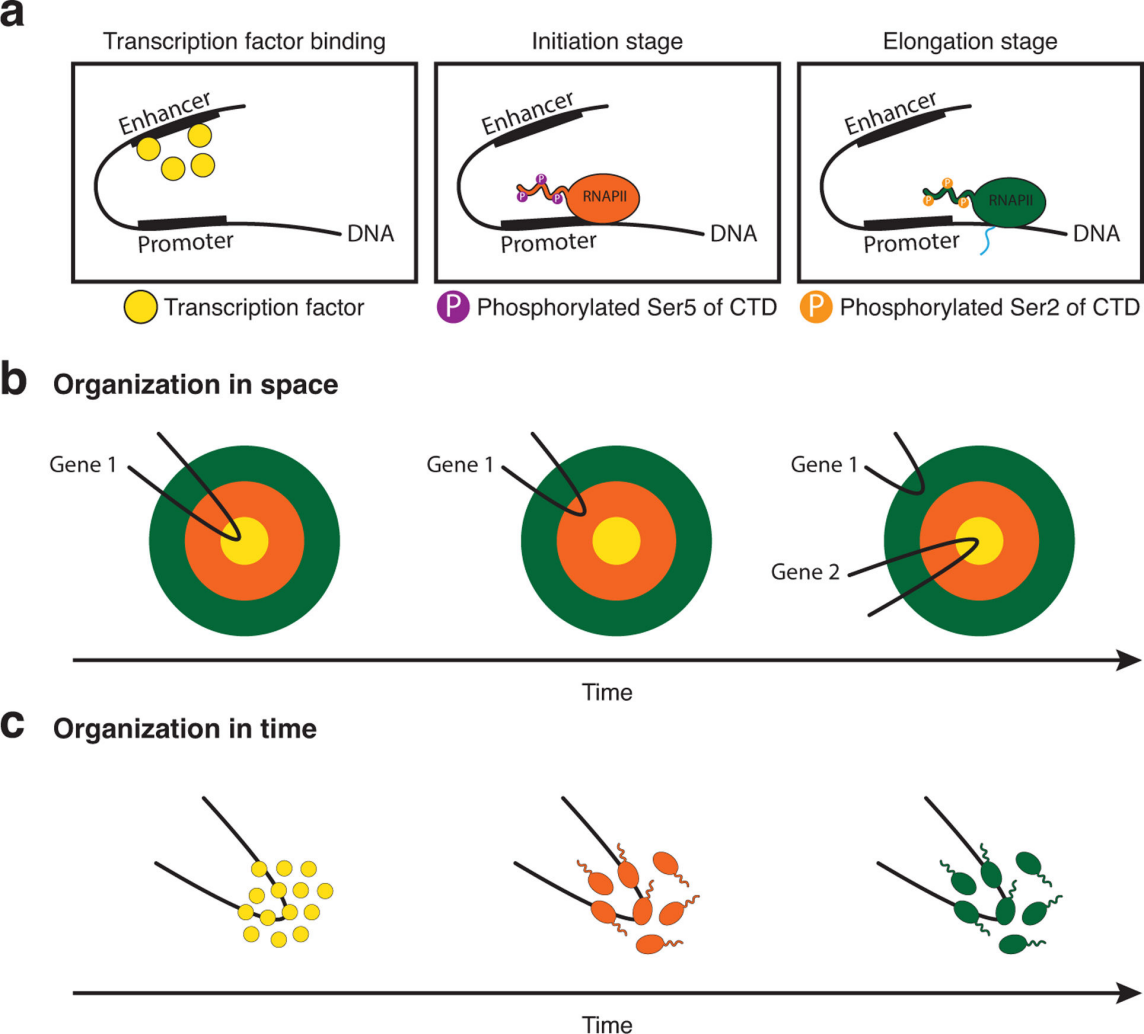
Organization of transcription bodies in space and time. **(a)** Schematic representation of transcription as a multistep process. **(b)** Organization of transcription bodies in space. **(c)** Organization of transcription bodies in time. See text for more detail.

The biochemical characterization of transcription has shown that the activation of a gene is a sequential process that requires many proteins to be recruited and modified in a well-defined order (Figure 2a). One way to organize such a sequential process in transcription bodies is *in space*. In this case, the different steps of transcription would occur in specific regions of the transcription body, with the gene moving from one region to the other while progressing through transcription (Figure 2b). This type of organization was observed for the nucleolus [25], a highly specialized transcription body in which the expression of most rRNAs and their assembly into ribosomal subunits occurs [26]. The structure of the nucleolus reflects the sequential assembly line that leads to ribosome synthesis, with radially ordered shells in which different stages of synthesis occur. Another way to organize a sequential process is *in time*. In this case, the transcription body would mature from a transcription factor cluster to being an initiating and then elongating transcription body (Figure 2c).

Because of the spatial organization of the nucleolus, it had been speculated that RNAPII transcription bodies could also be organized in space. Indeed, previous studies have suggested that a shell structure could also exist for transcription bodies, with an internal shell in which transcription initiation occurs, and an outer shell in which transcription elongation occurs [4,27]. While, in the analysis of fixed samples, temporal organization may be falsely perceived as spatial organization [4], there is also evidence for a spatial organization of transcription bodies using live imaging [28,29]. Here, super-resolution microscopy was used to show that in zebrafish transcription bodies, different genes can be expressed in subregions and that initiation and elongation can sometimes be spatially separated.

In contrast, studies that have followed the maturation process of transcription bodies over time in fly and zebrafish embryos have found that the sequential process of transcription is reflected in a maturation of the transcription body *over time* [23,30] (Figure 2c). In zebrafish embryos, the first two visible transcription bodies originate from DNA-bound Nanog clusters that then recruit Sox19b. These transcription factor clusters then recruit RNAPII, which transitions to the initiating state and finally the elongating state. Only in the elongating state, one can observe the transcripts of the genomic loci contained within the body [22,23,30,31]. The transcription bodies then disassemble toward the end of the cell cycle, when RNA has accumulated [23,30]. This is consistent with the observation that the accumulation of transcribed RNA can result in the dissolution of clusters [32]. Thus, these transcription bodies mature from a transcription factor cluster, to become an initiating and then elongating transcription body. These observations are supported by data from the fly, in which the transcription factor Zelda first forms transcription factor clusters that subsequently mature into active transcription bodies [33,34]. In these studies, histone acetylation was shown to be an important maturation step, as active RNAPII-containing transcription bodies depend not only on Zelda but also on the acetyltransferase CBP.

Taken together, a picture emerges in which the temporal maturation of transcription bodies appears to be more prevalent in early embryos, while the spatial separation of transcription initiation and elongation might be more common in more differentiated cells. A temporal maturation of the transcription process suggests a fundamentally different way of transcription regulation than a spatial organization. It predicts, for example, that genes come together to be transcribed, thereby nucleating a transcription body that will disassemble once the expression of these genes is completed. This is in line with the widely observed burst-like behavior of transcription [35]. A temporal maturation of transcription bodies also predicts that the genes in a body are expressed in a synchronous manner. In contrast, an increased reliance on spatial organization would suggest that bodies are stable structures toward which genes move to be expressed. This would be predicted to support the continuous expression of genes, as observed for housekeeping genes. This type of organization would allow genes to enter and leave the transcription body independently of each other and thus be transcribed in a nonsynchronous way.

We conclude that the organization of transcription bodies can occur in space or in time, and the type of organization affects transcription body function. The relationship between transcription bodies, genes, and transcription will be better understood in future studies that will characterize the interaction of transcription bodies with their target genes, something that has only recently become possible, thanks to improvements in live imaging of single-copy genes [36–38].

### Transcription bodies are important for developmental gene expression

It has been proposed that the local increase in protein concentration in clusters accelerates biochemical reactions [39]. As a consequence, transcription bodies have sparked a lot of interest as they might explain how transcription factors manage to find and bind their target sites in the genome. This is not a trivial problem, considering how densely packed the nucleus is, how big and folded the genome is, and how degenerate many binding motifs are. Indeed, it was estimated that a transcription factor would need hours to find its target site solely through diffusion in the three-dimensional space given these search conditions [40]. However, studies in cell culture have shown that transcription factors find their targets much faster [41]. Moreover, clustering has been shown to increase target search efficiency [42,43] and facilitate transcription [30,44,45]. Thus, at the molecular level, it appears that clustering of machinery can facilitate transcription. Embryonic systems have been key to showing that transcription factor clustering is relevant *in vivo* to regulate transcription.

**Figure 3:**
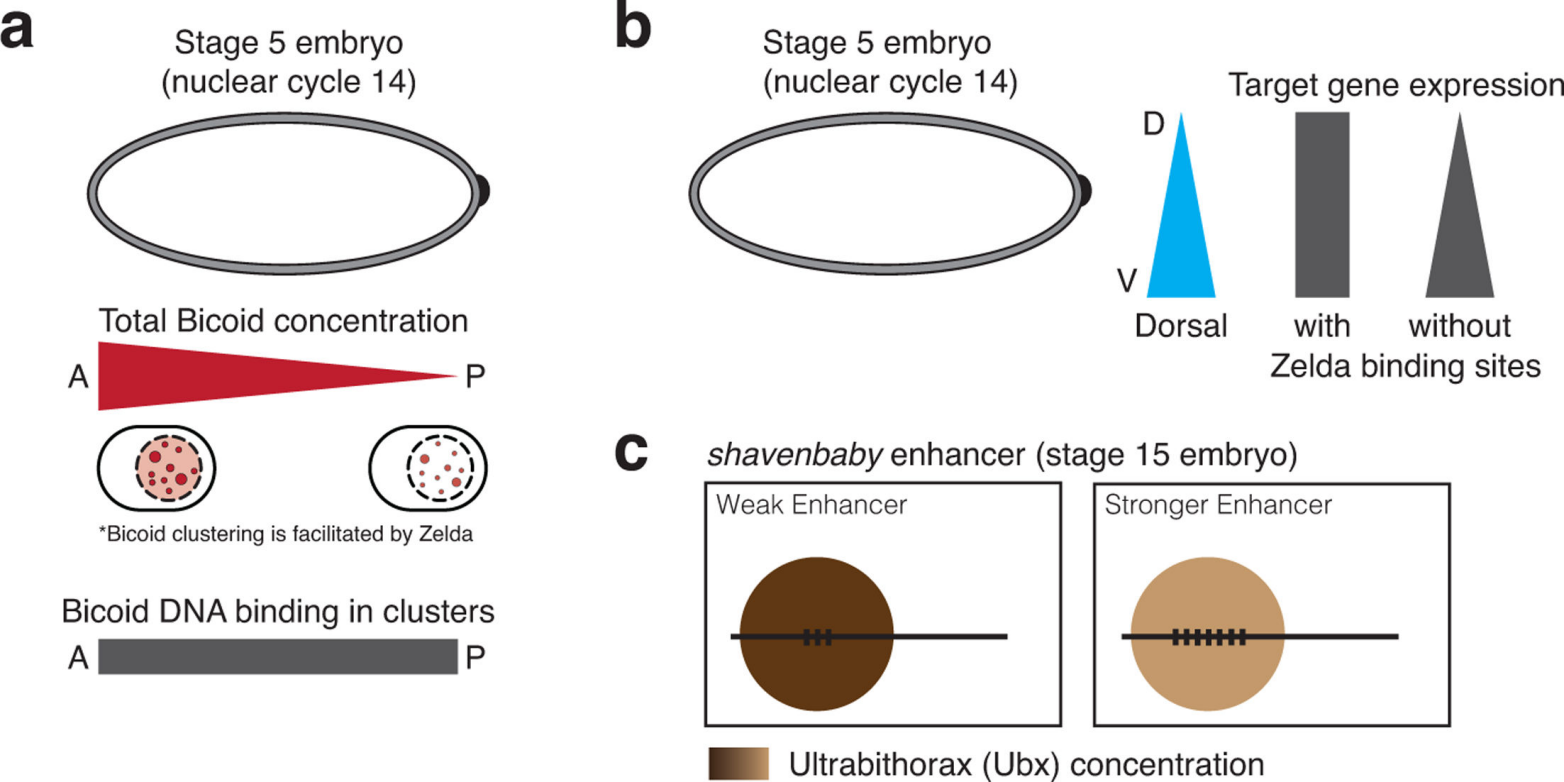
Transcription factor clustering can compensate for low transcription factor concentration and low enhancer affinity in *Drosophila* embryos. **(a)** The transcription factor Bicoid forms an anterior/posterior (A/P) gradient at stage 5 (nuclear cycle 14). Bicoid forms clusters at both poles, facilitated by Zelda [47]. The clustering at the posterior pole compensates for the low overall transcription factor concentration by maintaining a constant frequency of transcription factor binding events within the clusters [47]. **(b)** The transcription factor Dorsal forms a dorsal/ventral (D/V) gradient at stage 5 (nuclear cycle 14). Its clustering, which requires Zelda-binding sites [49], compensates for the decrease in overall concentration along the gradient, maintaining the expression of its target gene *sog* constant throughout the embryo [49]. **(c)** Low-affinity enhancers in the *shavenbaby (svb*) locus in the fly are often found in clusters of high Ultrabithorax (Ubx) concentration [50]. This is expected to increase rates and therefore increase transcription output. Upon artificially increasing the affinity of *svb* enhancers, they were found in lower intensity Ubx clusters [50].

The fly embryo has well-described transcription factor gradients (Figure 1d). Bicoid, for example, distributes along a decreasing concentration gradient along the anterior–posterior axis [46]. Despite low overall Bicoid concentrations at the posterior end, Bicoid target genes are expressed in this domain. Interestingly, Bicoid forms clusters [47,48]. These high-density areas facilitate binding when overall Bicoid levels are low by modulating transcription factor on rates (Figure 3a). The clustering of Bicoid depends on Zelda [47], and Bicoid-binding events are enriched in Zelda hubs [48]. This suggests the exciting possibility that Zelda facilitates the clustering of Bicoid, which leads to transcriptional activation, even when Bicoid levels are low [47]. Zelda plays a similar role in the clustering of the transcription factor Dorsal, which forms a dorsoventral gradient in the fly embryo (Figure 3b). Zelda buffers the concentration changes along the gradient by enhancing the clustering of Dorsal at low concentrations (dorsally), ensuring a constant expression of target genes like *sog* [49]. Thus, transcription factor clustering can compensate for low transcription factor concentration.

Similar to the ability of low transcription factor concentrations to activate transcription, low-affinity transcription factor binding sites are also capable of driving efficient gene expression. Here too, the fly embryo was used to understand this apparent contradiction. The *shavenbaby (svb*) locus in the fly is well characterized, and its expression is driven by the transcription factor Ultrabithorax (Ubx), which binds to a set of low-affinity binding sites in enhancers. Interestingly, these enhancers are often found in clusters of high Ubx intensity (Figure 3c) [50]. This is expected to increase rates and therefore increase transcription output. Thus, transcription factor clustering can compensate for low-affinity binding sites. Interestingly, when the affinity of *svb* enhancers was artificially increased, they colocalized with lower intensity Ubx clusters [50]. While these observations help explain how expression can be driven from low-affinity enhancers, they raise some important points. First, the association of clusters with low-affinity enhancers is counter-intuitive, as clustering tends to be driven by high-affinity sites [51,52]. This suggests that the Ubx clusters that are associated with low-affinity *svb* enhancers might be seeded by other (high-affinity) enhancers. It remains unclear, however, which enhancers seed these clusters, how the low-affinity *svb* enhancers know that they need to go there, and how they get there [53].

The above illustrates how the use of developmental models has revealed the relevance of transcription bodies in developmental gene expression.

### Transcription bodies sequester transcription machinery

The accumulation of machinery can have a local beneficial effect, but it may also affect the availability of machinery elsewhere by sequestering it. Probably the best-known example of sequestration in biology are lysosomes, cytoplasmic membrane-bound organelles that digest biomolecules. These organelles sequester lytic enzymes to protect the cell from their toxic effects. Other membraneless compartments have been shown, for example, to buffer concentration changes of the DEAD-box helicase DDX4 [54], to maintain genomic stability by sequestering Pumilio [55], and to inhibit apoptosis by sequestering caspases [56]. Regarding nuclear processes, the transcriptional repressor Daxx was found to be sequestered in specific bodies, thereby restricting its repressive effect [57].

**Figure 4:**
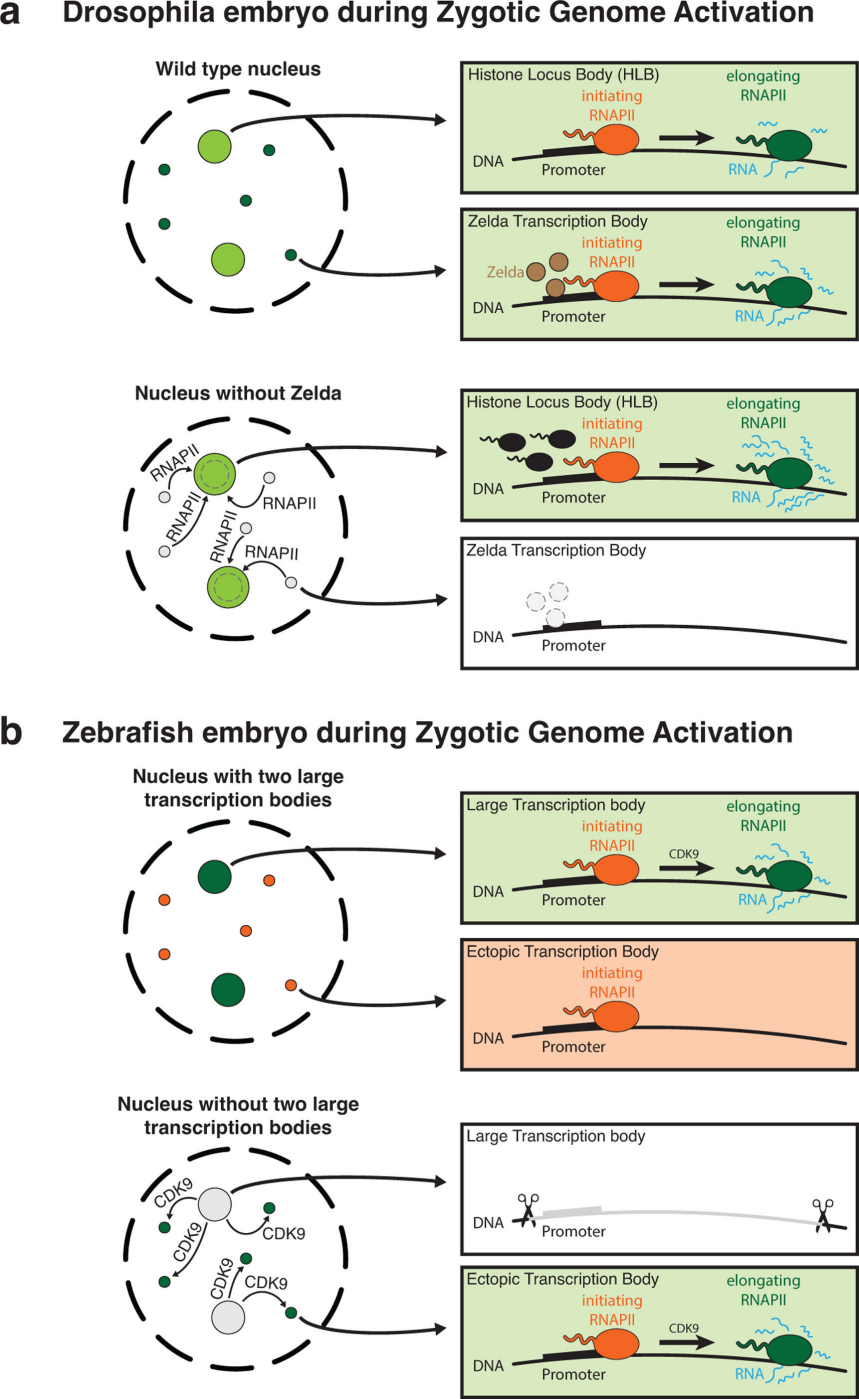
Transcription bodies sequester transcription machinery. **(a)** In fly embryos, Zelda-nucleated transcription bodies and histone locus bodies (HLBs) compete for transcription machinery like RNA polymerase II (RNAPII) [59]. Consequently, the disruption of Zelda-nucleated transcription bodies by deleting Zelda causes a redistribution of RNAPII toward the HLBs, which increases their size and transcriptional activity. **(b)** in zebrafish embryos, two large transcription bodies and many small ectopic transcription bodies compete for the transcription machinery involved in the transition from initiation to elongation, like CDK9 [22]. Consequently, the disruption of the two large transcription bodies by deleting the *mir430* locus (the nucleator sequence of the two large transcription bodies) causes a redistribution of CDK9 toward the ectopic transcription bodies, which makes a higher fraction of them enter the elongation phase.

Recently, work in fly and fish embryos, as well as in Arabidopsis, has revealed that positive regulators of transcription can also be sequestered [22,58,59]. In fruit fly embryos, for example, two major types of transcription bodies, namely Zelda-nucleated transcription bodies and the histone locus bodies (HLBs), compete for access to the transcription machinery. Indeed, without Zelda, the available transcription machinery redistributes toward the HLBs, increasing their size and transcriptional output [59] (Figure 4a). Similarly, work in mouse embryonic stem cells (mESCs) has shown that highly accessible loci can recruit transcription machinery to the point of down-regulating other genes [60].

The role of transcription bodies in sequestering transcriptional machinery has been investigated in more detail in zebrafish embryos. The two transcription bodies in which transcription is initiated during zebrafish genome activation are seeded by the *mir430* locus [23]. This knowledge allowed for the specific disruption of the transcription bodies, by the deletion of this genomic locus. An in-depth characterization of the mutant without transcription bodies revealed that in wild-type embryos, the two large transcription bodies accumulate CDK9, the kinase of the P-TEFb complex, to such an extent that other transcription bodies are deprived of CDK9 activity and remain stalled at the initiating state. Indeed, the specific disruption of the two large transcription bodies freed up the sequestered CDK9, allowing the remaining transcription bodies to complete their maturation process and become elongating transcription bodies [22] (Figure 4b). Importantly, the overexpression of CDK9 phenocopied this behavior.

It is important to point out that this novel function of transcription bodies in sequestering transcription machinery was discovered in zebrafish embryos that have two isolated and large transcription bodies. Disrupting these two transcription bodies, therefore, had a large enough effect on the transcriptome that it could be measured. In contrast with this measurable effect, a similar manipulation in cell culture, where transcription bodies are much smaller and more abundant, would have most likely caused an undetectably small perturbation, if machinery is even sequestered to a significant extent in this system. In conclusion, the use of developmental models has allowed us to identify a novel and previously underappreciated role of transcription bodies in transcription, highlighting the complex role these structures have in gene expression regulation.

**Future perspectives**

- Transcription bodies are nuclear structures in which transcriptional machinery accumulates and transcription takes place. More knowledge of their form and function is needed to improve our understanding of transcription regulation in development and disease.
- Studies in embryos have revealed that transcription bodies (i) can be organized in time, as well as in space, (ii) are important for developmental gene expression by compensating for low transcription factor concentration and low-affinity enhancers, and (iii) can regulate transcription factor availability by sequestration.
- Technical advances in low-input omics technologies and high-resolution imaging will further increase the benefits of developmental model systems in the future, for example, in delineating how regulatory elements interact with transcription bodies, which will be required to fully understand transcription body function.

## Open Access

This article has been published open access under our Subscribe to Open programme, made possible through the support of our subscribing institutions, learn more here: https://portlandpress.com/pages/open_access_options_and_prices#conditional

## Abbreviations

A/P: anterior/posterior
D/V: dorsal/ventral
HLBs: histone locus bodies
RNAPII: RNA polymerase II
TF: transcription factor
Ubx: Ultrabithorax
ZGA: zygotic genome activation
mESCs: mouse embryonic stem cells
svb: shavenbaby
